# Assessment of genetic diversity and inbreeding in the Okinawa indigenous Agu pig through pedigree analysis

**DOI:** 10.5713/ab.24.0646

**Published:** 2025-02-27

**Authors:** Shihei Touma, Kyota Fusho, Aisaku Arakawa

**Affiliations:** 1 Okinawa Prefectural Farming Support Department, Naha, Okinawa, Japan; 2 Okinawa Prefectural Livestock and Grassland Research Center, Nakijin, Okinawa, Japan; 3 Institute of Livestock and Grassland Science, National Agriculture and Food Research Organization (NARO), Tsukuba, Japan

**Keywords:** Agu Population, Effective Population Size, Genetic Diversity, Inbreeding, Indigenous Pig, Pedigree Analysis

## Abstract

**Objective:**

The objective of this study was to clarify the changes in genetic diversity and inbreeding over the years as well as to identify the causes of genetic diversity loss using the pedigree of Okinawa indigenous Agu pigs.

**Methods:**

The pedigree of the Agu population consisted of 1,997 animals from 1997 to 2020. The equivalent complete generations (ECG), inbreeding coefficient (F), and effective population size (Ne) were computed using ENDOG v4.8. Genetic diversity parameters, including founders (*f*), the effective number of founders (*f_e_*), the effective number of ancestors (*f_a_*), and the founder genome equivalent (*f_ge_*), were derived from the probability of gene origin. The study also investigated changes in genetic diversity indices over time.

**Results:**

ECG increased from 0.1 in 2003 to 3.87 by 2020, indicating an improved pedigree depth. The F has been on a downward trend since it peaked at 10.5% in 2015. However, the N_e_ in the reference population was 14.6, which is below the recommended levels. The *f*, *f_e_*, *f_a_* and *f_ge_* of the reference population were 86, 26, 19, and 11.5, respectively. These values decrease in the order of *f*>*f_e_*>*f_a_*>*f_ge_*. This indicates that all factors of genetic diversity loss played a role, including the unbalanced contributions of founders, bottlenecks, and random gene losses. Although in recent years efforts have been made to maintain genetic diversity and reduce inbreeding, the Agu pig population exhibited a 5% decrease in genetic diversity over the past 18 years, with both unequal founder contributions and genetic drift identified as significant contributing factors.

**Conclusion:**

This study reveals that more favorable breeding strategies (such as optimal contribution and population size expansion) are required to maintain genetic diversity and increase the N_e_ of the Agu pig population.

## INTRODUCTION

Indigenous and local animal breeds are typically reared in unique environments and have usually adapted to the local environments. Some of these breeds serve as a source of high-quality products [1], but they are usually less productive than the improved commercial breeds. Therefore, since the mid-20th century, high-performance breeds have become prevalent worldwide, often replacing indigenous and local breeds [2]. As a result, 14% of pig breeds worldwide are at risk of extinction [3], thus prompting efforts to conserve local pig breeds [4–8].

Agu pigs have been reared in the Okinawa Prefecture, which is the southern- and westernmost prefecture of Japan. The Agu population size was previously over 100,000 but exhibited a sharp decline because of the introduction of European breeds in the early 1900s [9]. In the 2000s, Agu pork was evaluated by consumers to have high meat quality, and its demand quickly increased. Around the same time, conservation programs for the Agu population started to recover the genetic diversity. In 2005, the Okinawa Prefecture government and the Okinawa Prefecture Livestock Improvement Association conducted a pedigree survey to reconstruct the Agu breed and established a registration system for the Agu population. Nowadays, Agu pigs hold high socio-cultural value as the only indigenous pig in Japan, and Agu pork production has become an important agricultural production system in Okinawa because of its excellent meat quality [10,11].

Pedigree analysis has been used to monitor genetic diversity over generations in both commercial [12–14] and local breeds [5–8]. It has been more than a decade since the conservation program for the Agu population began, and sufficient pedigree information has been collected, making it possible to evaluate the genetic diversity of the population with high accuracy. This study aimed to assess the change in genetic diversity and inbreeding over the years and to identify the main causes relating to the loss of genetic diversity using pedigree information of the Agu population.

## MATERIALS AND METHODS

### Pedigree data and reference population

**Figure 1:**
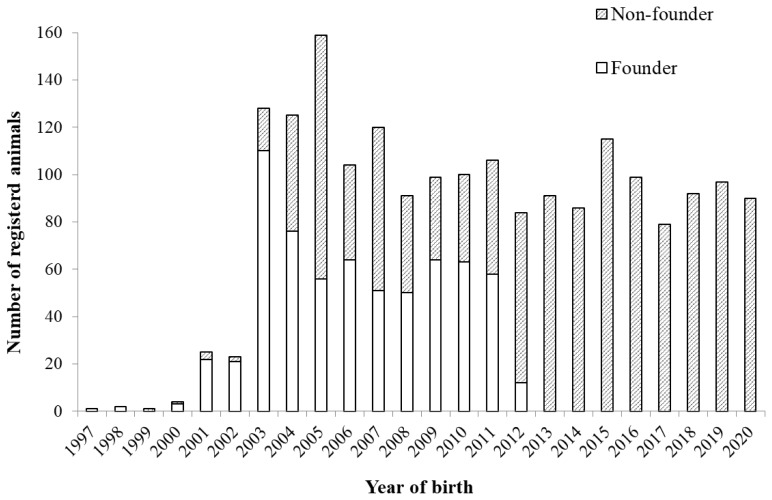
Number of animals registered as breeding females and males per year of birth.

The pedigree information consisted of 1,997 breeding animals that were born from 1997 to 2020, which was provided by the Okinawa Prefecture Livestock Improvement Association. Animals with both parents that are unknown were regarded as founders. Figure 1 shows the number of breeding animals in each birth year. When the registration system for Agu pigs was initially implemented individuals without parental information were allowed to be registered as founders. Agu registration applications with no parent information were closed in March 2012. As a result, the birth of founders was predominant until 2011, but in 2012, the number of founders decreased drastically. Since 2013, all newly born animals have pedigree information, meaning that the Agu population has been maintained in a closed system. In this study, animals born between 2003 and 2020 were used for the analysis, and the reference population was defined as individuals born from 2017 to 2020. The reference population consisted of 358 animals, including 202 females and 156 males (Table 1).

### Pedigree quality

The completeness and depth of the pedigree information were assessed using the pedigree completeness index (PCI) and equivalent complete generations (ECG). For each animal, the PCI was calculated as:


$$PCI=\frac{4I_{sire}I_{dam}}{I_{sire}+I_{dam}}$$

where *I_sire_* and *I_dam_* are the indices for paternal and maternal contribution, respectively, and


$$I=\frac{1}{d}\sum_{i=1}^{d}a_{i}$$

where *a_i_* is the percentage of ancestors known in generation *i* and *d* is the number of generations. ECG was calculated as the sum of all known ancestors and was computed using the following formula:


$${ECG}_{i}=\sum {\left(\frac{1}{2}\right)}^{n}$$

where *n* is the number of generations between the individual and each known ancestor and the sum is computed across all known ancestors of the *i*th animal [15].

### Inbreeding and effective population size

The inbreeding coefficient (F) was computed according to the algorithm previously described by Meuwissen and Luo [16], and the coancestry coefficient (C) was calculated using the algorithm described by Colleau [17]. The estimate of effective population size (*N_e_*), also called the realized *N_e_*, was calculated as:


$$N_{e}=\frac{1}{2\bar{\Delta F}}$$

where $\bar{\Delta F}$ is the mean value of the increase in inbreeding (Δ*F_i_*) in a given reference population. Δ*F_i_* was computed using the approach previously proposed by Gutiérrez et al [18,19] as:


$$\Delta F_{i}=1-\sqrt[{t-1}]{1-F_{i}}$$

where *F_i_* and *t* represent the coefficient of inbreeding and ECG, respectively, for an *i*th individual.

### Parameters of genetic diversity

To measure the genetic diversity of Agu pigs, parameters based on the probability of gene origin (effective number of founders [*f_e_*], effective number of ancestors [*f_a_*], and founder genome equivalent [*f_ge_*]) were used. The *f_e_* was calculated using the following formula:


$$f_{e}=\frac{1}{\sum_{i=1}^{f}p_{i}^{2}}$$

where *p_i_* is the genetic contribution of the *i*th founder to the reference population and *f* is the total number of founders. *p_i_* is calculated as follows:


$$p_{i}=\frac{g_{i}}{N}$$

where *g_i_* is the sum of the genetic relatedness between founder *i* and animals in the reference population and *N* is the number of animals in the reference population. *f_e_* is a measure of the founder’s contributions to the reference population and is defined as the number of founders that equally contributed to the reference population [20]. If all founders contribute equally to the reference population, then *f_e_* is equal to *f*, but if the genetic contribution of the founders is unequal because of selection, then *f_e_* is smaller than *f*.

The *f_a_* was calculated to identify population bottlenecks using the following formula [21]:


$$f_{a}=\frac{1}{\sum_{i=1}^{a}q_{i}^{2}}$$

where *q_i_* is the marginal contribution of the *i*th ancestor to the reference population and *a* is the number of influential ancestors. The marginal contribution of an ancestor is the expected ancestral contribution with redundant contributions of the other ancestors being eliminated, and *q_i_* is calculated using the following equation:


$$q_{i}=p_{i}\left(1-\sum_{k=1}^{n-1}x_{k}\right)$$

where *x_k_* is the expected genetic contribution of the *n*−1 selected ancestors to the *k*th animal. For further details and explanations of the marginal contributions, we recommend referring to Boichard et al [21]. *f_a_* is the minimum number of ancestors, not necessarily founders, which explains the complete genetic diversity of the population under study.

The *f_ge_* can be defined as the number of equally contributing founders that would be expected to provide the same genetic diversity as in the reference population if no loss of alleles occurred [20,22]. *f_ge_* was calculated as:


$$f_{ge}=\frac{1}{2\bar{C}}$$

where *C̄* is the average coancestry of the individuals included in the reference population, expressed using the following equation:


$$\bar{C}=\frac{\sum_{i=1}^{N}\sum_{j=1}^{N}A_{ij}}{2N^{2}}$$

where *A_ij_* is the additive relationship coefficient between animals *i* and *j*.

Genetic diversity measures can be derived from *f_e_* and *f_ge_*. The total amount of genetic diversity in the reference population (*GD*) that can explain the loss of genetic diversity due to genetic drift and unequal founder contributions, was calculated as follows [22]:


$$GD=1-\frac{1}{2f_{ge}}$$

The value of 1−*GD* is an indicator of the genetic diversity loss of the reference population caused by bottlenecks and genetic drift that occurred in the generations after the founders. The amount of genetic diversity loss of the reference population caused by the contribution of unequal founders (GD*) was calculated as follows [22]:


$$GD^{*}=1-\frac{1}{2f_{e}}$$

The loss of genetic diversity because of an unequal number of founders was expressed as 1−GD* [23]. Furthermore, GD*−*GD* represents the loss of genetic diversity due to random genetic drift.

All parameters described above were computed using ENDOG v4.8 software [24].

## RESULTS

### Pedigree completeness and generation depth

According to the changes in the number of breeding animals by birth year, the number of animals exhibited a slight decreasing trend after peaking in 2005 but remained relatively stable at approximately 100 individuals (Figure 1). The proportion of known ancestors was 100% in the first generation and decreased with each subsequent generation, dropping as low as 23% in the fifth generation (Table 1).

**Figure 2:**
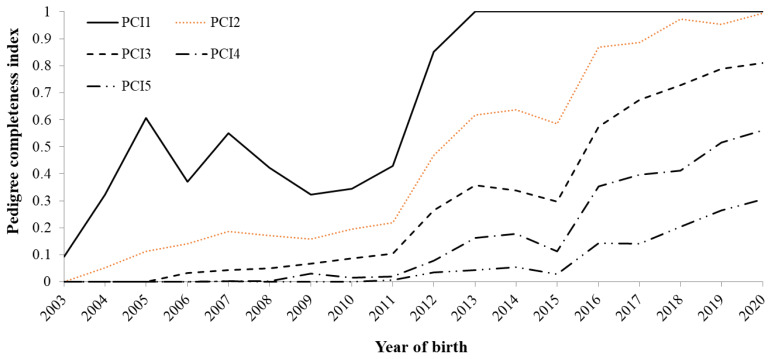
Pedigree completeness index (PCI) from accounting for 1 (PCI1) to 5 (PCI5) generations back in Agu pigs.

**Figure 3:**
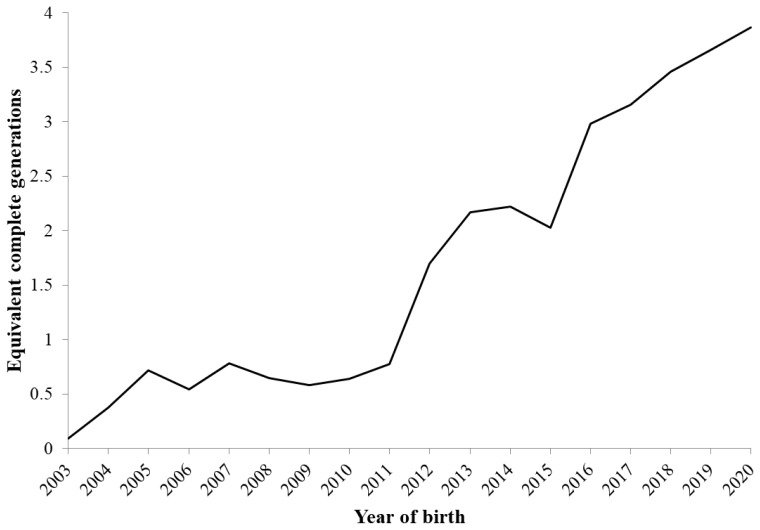
Changes of equivalent complete generations (ECG) in Agu pigs.

Figure 2 shows the change in the average PCI over the birth years, accounting for 1 to 5 generations back for all animals. The PCI values increased from 2012 to 2020, where PCI1 achieved 100% in 2013, and PCI2 was >90% in 2018. The ECG remained below 1 until 2011 because new animals that had unknown pedigree information were added to the population, and finally, in 2020, it reached 3.87 (Figure 3).

### Inbreeding and effective population size

**Figure 4:**
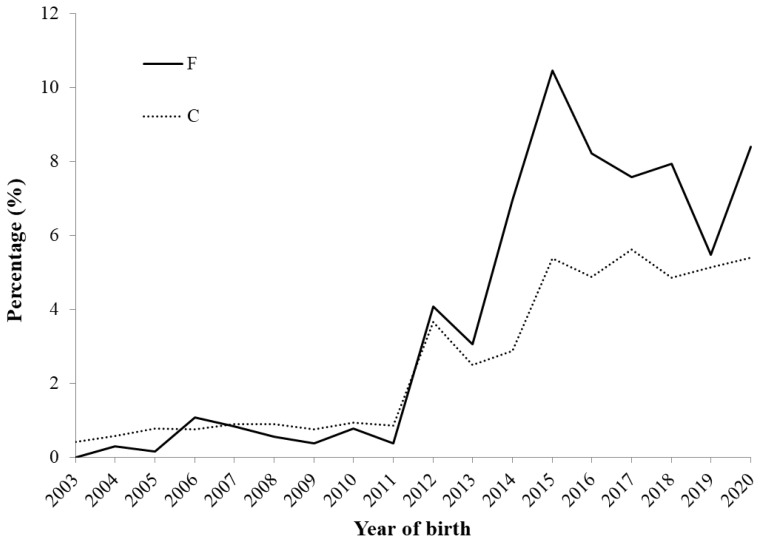
Changes of average inbreeding (F) and coancestry (C) in Agu pigs.

**Figure 5:**
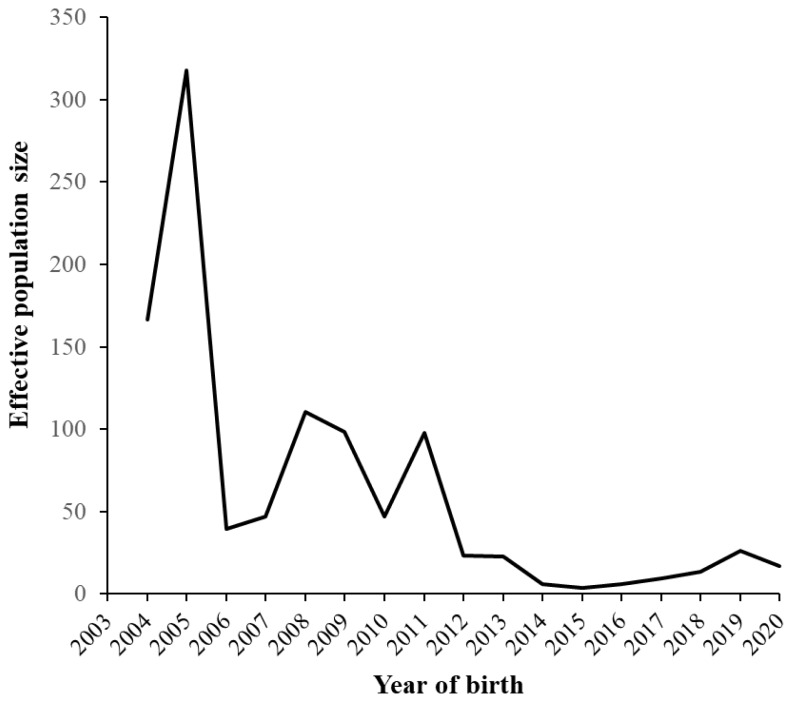
Changes of effective population size (Ne) in Agu pigs.

Changes in the F and C values are shown in Figure 4. The F values began to rapidly increase in 2012, reaching a peak of 10.5% in 2015, and then they exhibited a decreasing trend. The high F value in 2015 was because the male that produced the most offspring that year (33 out of 115 born) who mated with a closely related female, resulting in offspring with elevated F values. These 33 offspring had an average F value of 27.3% (ranging from 25% to 37.5%). The C values showed a similar trend to F, but the increase rate was relatively gradual. In recent years, it has remained stable at approximately 5%. The C values have been lower than the F values since 2012, and the difference between them has been increasing. The average F value in the reference population was 7.3%, while the ΔF and N_e_ values were 3.4% and 14.6, respectively (Table 2). The annual changes in N_e_ are shown in Figure 5. From 2003 to 2005, N_e_ was above 150, and from 2006 to 2011, it ranged approximately between 50 and 100. However, since 2012, it has consistently been below 50.

### Probabilities of gene origin and genetic diversity

**Figure 6:**
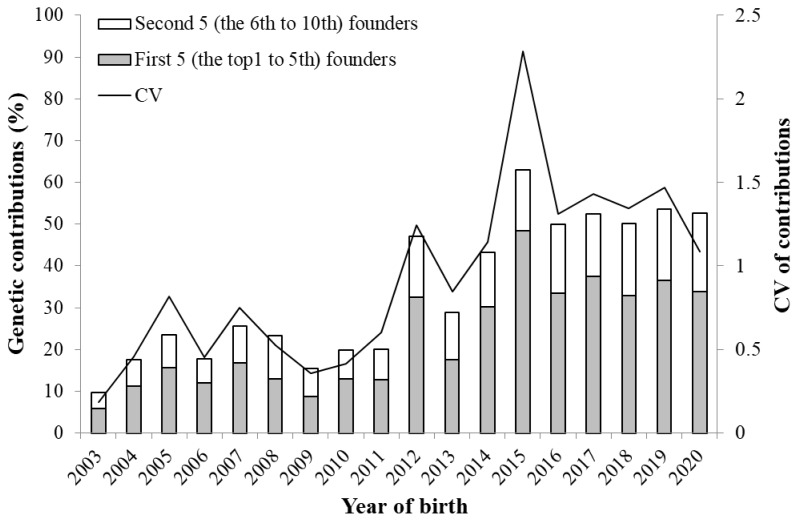
Cumulative genetic contributions of the top 10 representative founders (left axis), CV of genetic contribution of all founders (right axis). CV, coefficient of variation.

**Figure 7:**
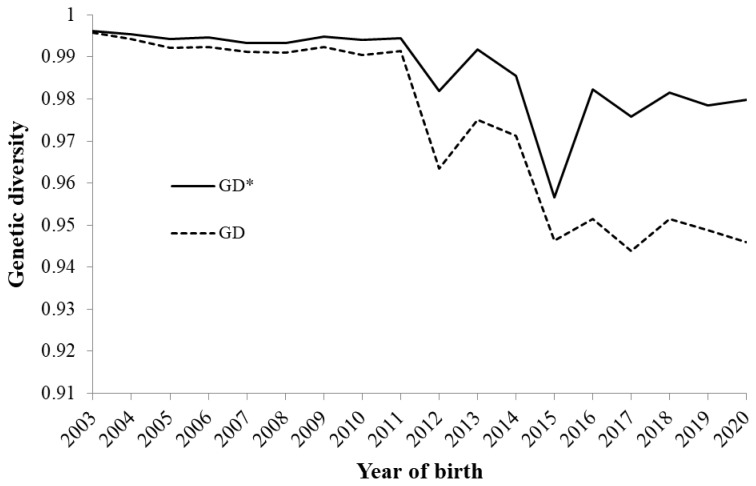
Changes in genetic diversity accounting for loss of diversity due to unequal founder contribution and random genetic drift (GD) and due to only to unequal founder contribution (GD*) in Agu pigs.

Table 3 shows the parameters derived from the probability of gene origin within the reference population (2017–2020); the parameters decreased in the following order *f* >*f_e_*>*f_a_*>*f_ge_*. The ratios of *f_e_* / *f* and *f_e_* / *f_a_* were 0.3 and 1.37, respectively. The gene pool of the reference population accounted for 50% of the eight ancestors. Figure 6 shows changes in the cumulative values of the genetic contributions of the first five (the top 1st to 5th) and second five (the 6th to 10th) founders as well as the coefficient of variation (CV) of the genetic contributions of all founders. The increase in the contributions since 2012 resulted in an increase in the unequal contribution of the founders during that time, and the genetic contribution of the first five founders accounted for >30% in recent years. In 2015, the CV was higher than the other years, which was caused by the genetic contribution of the one founder (male) that accounted for 25.4% of individuals born. As a result, the inequality in the genetic contribution of the founders increased. Figure 7 shows the change in the two genetic diversity indices (GD* and *GD*) over 18 years. Both the indices exhibited a sharp decline in 2012, but since 2016, the GD* and *GD* values remained at approximately 0.95 and 0.98, respectively. The total loss of genetic diversity (1−*GD*) over the 18 years was approximately 5%, of which 2% of this was due to unequal founder contribution (1−GD*) and the remaining 3% was due to genetic drift (GD*−*GD*).

## DISCUSSION

Similar to the population of Agu pigs, approximately 29% of local pig breeds maintain a small population, and thus, their genetic diversity is quite low, with a high endangered risk [25]. In the Agu population, there were only 171 breeding females in 2020, classifying them as endangered (100 to 1,000 breeding females) according to the Food and Agriculture Organization’s (FAO’s) risk-status classification [3]. A small population size results in the loss of genetic diversity and the rapid increase of inbreeding, which eventually causes an inbreeding decrease in fitness-related traits [26]. A previous study using microsatellite markers has shown a higher degree of inbreeding in Agu pigs based on the observed heterozygosity compared to one of the modern European breeds [9]. Meanwhile, pedigree information can provide good indicators to monitor a population’s genetic diversity, and this approach is more cost-effective than molecular analyses [27], especially in the case of local breed conservations where financial support is generally limited. The information allows for the assessment of genetic diversity and demographic parameters across generations, thus providing insight into unbalanced founder contributions and genetic drift.

In this study, the quality of the Agu pedigree accumulated from the beginning of the Agu registration, the change in genetic diversity over the years, and the inbreeding parameters of the Agu population were investigated. The ECG value was 3.55 in the reference population (Table 1), which was lower compared to other local breeds. For instance, the ECG for Nero di Parma in Italy was 7.22 [8] and 4.38 for the Bísaro pig in Portugal [7]. Regarding the PCI, Melka and Schenkel [12] investigated the PCI accounting for four generations back (PCI4) of the Canadian Duroc, Landrace, Yorkshire, Lacombe, and Hampshire breeds, and they reported that Hampshire had the lowest PCI at 52.7%. While Agu’s PCI4 was even lower at 48%, the overall depths of pedigree in Agu pigs were small. This is because a substantial number of individuals had unknown pedigree information until relatively recently, which resulted in shallower pedigree depths.

The F and C values increased rapidly from 2012 to 2015 (Figure 4). During this period, most farms had adopted a closed population, corresponding to the beginning of an increase in ECG (Figure 3), which influenced the large increase in the F and C values from 2012 to 2015. Generational turnover within the small populations of Agu pigs has likely increased the genetic relatedness among individuals. An average C value between parents equals the F value of their progeny [28], and if undergoing random mating, the average C value in a generation is expected to be higher than the F value in the subsequent generation. However, since 2012, the F values have been higher than the C values, which suggests that non-random mating has been performed because of the limited exchange of Agu breeding stocks among farms and a lack of appropriate mating strategies. To reduce the increase of inbreeding, the Okinawa prefectural government has guided appropriate mating to avoid inbred mating and has distributed semen and breeding animals from prefectural farms, which are government-managed facilities that maintain breeding animals with lower genetic relatedness to Agu pigs on other farms. Therefore, since 2016, the F values have exhibited a decreasing trend.

The F value in the reference population was 7.3% (Table 2), which is similar to other local breeds. For example, the three Mangalica breeds ranged from 4.07% to 5.87% [5], and one of the Bísaro pigs was reported to be 8.58% [7], whereas in Gochu Asturcelta pigs, the value was 24.9% [6]. F and C values calculated from the pedigree information generally depend on the quality and depth of the pedigree, and therefore, we could not easily compare our results with other studies. Especially because the pedigree information of the Agu population is shallow. For our study, the increasing rate of inbreeding (ΔF) is a more important parameter than the F value for genetic conservation programs [29]. The FAO [29] recommends that ΔF should be kept within 1% to minimize the negative effects of inbreeding, which corresponds to N_e_>50. In this study, the N_e_ values have consistently been below 50 since 2012. Specifically, in the reference population, ΔF and N_e_ were 3.4% and 14.6, respectively. Therefore, additional conservation plans to sustain the Agu population are needed.

The probabilities of gene origin are valuable tools for assessing the genetic diversity within breeds, even when considering only a few generations [20]. Therefore, this can be an effective indicator to maintain the Agu population, even with a shallow pedigree depth. The parameters decreased in the order of *f* > *f_e_* > *f_a_* > *f_ge_* (Table 3). The *f_e_* / *f* ratio shows the degree of genetic loss because of the unequal genetic contribution of the founders, and the ratio of the Agu population was 0.30, which is in agreement with other local breeds’ studies; that of the Nero di Parma pigs was 0.27 [8], and the three Mangalica breeds ranged from 0.30 to 0.31 [5]. The genetic loss due to the unequal genetic contribution of the founders could occur as a consequence of the excessive use of some animals as parents for subsequent generations [12]. The *f_e_* / *f_a_* ratio indicates a loss of genetic diversity due to bottlenecks [30], which should ideally be 1.0, where the higher the value, the greater the loss of genetic diversity. The *f_e_* / *f_a_* value of the Agu population was 1.37, which was higher than that of Bísaro (1.12) [7] and Nero di Parma (1.0) [8] pigs. The repetitive utilization of certain individuals with high reproductive performance as parents was considered to have resulted in the bottleneck of the Agu population. This is supported by the observation that only eight individuals account for 50% of the gene pool of the reference population (Table 3), with the top contributor, a specific ancestor, making a substantial contribution of 16.3% (data not shown). In addition, the *f_ge_* value in the Agu population was lower than *f_a_*, indicating a loss of genetic diversity because of the random loss of genes during segregation. Thus, the loss of genetic diversity in the Agu population was caused by the unequal genetic contributions of the founders and genetic drift (bottleneck and random gene loss).

The total loss of genetic diversity (1−*GD*), accounting for both unequal founder contributions and genetic drift, amounted to approximately 5% over 18 years (Figure 7), which was significantly higher than that of Bísaro pigs (0.81%) [7]. The unequal founder genetic contribution (1−GD*) and genetic drift (GD*−GD) attributed to 2% and 3% of the amount of the genetic loss, respectively. In general, genetic drift is the main cause of genetic diversity loss within small populations [31], but in the Agu population, although the influence of genetic drift was substantial, the impact of unequal founder contributions was also relatively large. Furthermore, the two indices of genetic diversity (GD* and *GD*) exhibited a remarkable decline after 2012. This is partly due to the advancement of generations (Figure 3), which accelerated genetic drift. In addition, the lack of introduction of new founders into the population greatly contributed to the increased inequality of founder contributions. Although the excessive use of certain founders as parents of subsequent generations induced unequal founder contributions even before 2011, the introduction of 641 new individuals with unknown parents into the Agu population prevented the genetic diversity from reducing through unequal contributions (Figure 1). As few new founders were introduced into the population since 2012, the inequality of genetic contributions has become more apparent, which was confirmed by the rapid increase in the CV of the founders’ contributions from 2012 to 2015 (Figure 6). On the other hand, since 2016, the GD* has increased to 0.98, and the *GD* has remained at 0.95, indicating that efforts to sustain genetic diversity for the Agu population have been effective.

The results of the study revealed that the genetic diversity has reduced by 5% over 18 years because of the unequal genetic contribution of the founders and genetic drift. Strategies to sustain the genetic diversity of the Agu population, such as increasing the N_e_ in Agu pigs, are needed. The optimal contribution strategy based on minimizing the average coancestry and ΔF leads to an increase in N_e_ [1,22,23]. Thus, the application of this strategy could be an option to sustain the Agu population. Fortunately, Agu pork has been established as a branded pork production system [11]. Increasing the number of individuals is likely to be profitable and sharing information regarding the current state of genetic diversity with producers could potentially facilitate the expansion of the breeding population.

## CONCLUSION

This study demonstrated that the genetic diversity of the Agu population has decreased by approximately 5% over the past 18 years because of unequal founder contributions and genetic drift. The inbreeding coefficient rapidly increased from 2012 to 2015, and thereafter the parameter reduced. Meanwhile, the N_e_ value is too small to sustain the population steadily, and therefore, effective conservation strategies (such as optimal contribution and expansion of the population size) are required to maintain the genetic diversity of the population.

## Figures and Tables

**Table 1:** Summary of the reference population (2017–2020)

Items	
Animals in the whole pedigree file	1,997
Animals in the reference population	358
Females	202
Males	156
ECG	3.55
% known ancestors at generation (PCI)	
1	100
2	96
3	76
4	48
5	23

ECG, Equivalent complete generations; PCI, pedigree completeness index.

**Table 2:** Based on individual increase rate in inbreeding in reference population (2017–2020)

Parameters	Values
C (%)	4.4
F (%)	7.3
ΔF (%)	3.4
N_e_	14.6

C, average coancestry; F, average inbreeding; ΔF, mean value of individual increase rate in inbreeding; Ne, effective population size.

**Table 3:** Parameters derived from the probability of gene origin in the reference population (2017–2020)

Parameters	Values
Number of founders (*f*)	86
Effective Number of founders (*f_e_*)	26
Effective Number of ancestors (*f_a_*)	19
Founder genome equivalent (*f_ge_*)	11.5
*f_e_* / *f*	0.30
*f_e_* / *f_a_*	1.37
Number of ancestors explaining 100%	78
Number of ancestors explaining 50%	8
